# Atherosclerosis and Atheroma Plaque Rupture: Normal Anatomy of Vasa Vasorum and Their Role Associated with Atherosclerosis

**DOI:** 10.1155/2014/285058

**Published:** 2014-03-20

**Authors:** Zhonghua Sun

**Affiliations:** Discipline of Medical Imaging, Department of Imaging and Applied Physics, Curtin University, GPO Box U1987, Perth, WA 6845, Australia

## Abstract

Atherosclerosis is primarily a degenerative disorder related to aging with a chronic inflammatory component. There are differences in expression among different vascular beds, inflicting a range of vascular diseases. The majority of studies focus on the inner and medial vascular layers, which are affected at the development of atherosclerosis. Recent evidence shows that the outer layer of blood vessels, composed of the adventitial layer and the vasa vasorum, not only plays a significant role in maintaining vessel integrity, but also reacts to atheroma. What is not clear is the extent of contribution of the outer layer to the process of atherosclerosis. Is it involved in the initiation, progression, and clinical expression of atheroma? Is the inflammation associated with atheroma limited to being merely reactive or is there a proactive element? This paper provides an overview of the normal anatomy of vasa vasorum and potential mechanism of plaque formation due to vascular injury (vasa vasorum) and microhemorrhage.

## 1. Introduction

There has been a slow but relentless interest in the role of the vasa vasorum, the anatomy of their origins, and their branch distribution due to their possible role in atherogenesis [1, 2], coronary interventions [3, 4], and in response to risk factors for atherosclerosis [5, 6]. Should atherogenesis be shown to be emanating primarily from the vasa vasorum and not via the intima lining the lumen, then this will be a paradigm shift in our thinking of atherosclerosis. It is a plausible hypothesis that would explain much, including many of the complications of diabetes mediated through microvessels.

Cardiovascular disease is the major cause of mortality and morbidity in developed countries and its prevalence is increasing in developing countries, and atherosclerosis is responsible for many of the severe manifestations, including myocardial ischemia, acute myocardial infarction, heart failure, and stroke. Atherosclerosis is the main cause of amputation in populations not at war. It is implicated in aortic and other major vessel diseases and their potential for adverse events. During plaque development many proangiogenic pathways are reactivated and this leads to formation of immature blood vessels prone to rupture [7]. Infiltration of microvessels into the media, intima, and plaques originates primarily from proliferating vasa vasorum (the microvessels supplying the major arteries). Known statistically proven risk factors for the development of atherosclerosis are well published and can be classified into precursors and accelerators [8]. High blood pressure is a central risk factor for those genetically predetermined and an accelerator in all causes of hypertension. Vast amounts of money are committed to its control and the cost benefit could be improved.

It is well-known that an inflammatory process occurs within the arterial wall at the site of a developing plaque [9–11], and the mechanism of injury to the intima and lipid infiltration of the media is the accepted atherogenic pathway, which is considered reactive to the atheroma. There is evidence that flow shear stress arises from disturbed flow from protruding plaque with vacuolation and turbulence that cause further intimal disruption, and this has been suggested as a mechanism for excess blood lipids to fuel deposition into plaques from the lumen of the vessel.

The hypothesis of the initiating process starting in the wall of the artery and the injury healing cycle in a vulnerable site providing the ongoing stimulus that perpetuates the formation of the atherosclerotic plaques deserves investigation. The accepted mechanisms for atherogenesis do not explain why atheroma is uniquely an arterial lesion not involving veins unless they are arterialised; that is, it is not a disease of the young and if we all live long enough each one of us will develop an atheromatous lesion. It is a disease in predictable sites that are clearly stress sites. Pulsatile continuous pressure, not flow, is the peculiar parameter related to arterial disease. Compliance, capacitance, and water-hammer effects are conspicuous only by their absence in the arguments for atherogenesis but are likely to be key associates of blood pressure forces. Better understanding of the mechanisms of atherogenesis is the key to improving prophylactic and management options based on the understanding of the physics and science of a physical parameter, that is, central to the disease and its management.

Plaque angiogenesis has been accepted to play a fundamental role in the pathophysiological development of atherosclerosis, providing nutrients to the developing and expanding intima and also potentially creating an unstable hemorrhagic environment prone to rupture [12, 13]. The expression of intimal neovessels is directly related to the stage of plaque development, the presence of symptomatic disease, and the risk of plaque rupture. Angiogenesis is involved in the development of atherosclerosis and associated with clinical syndromes in the coronary circulation and in the context of symptomatic carotid occlusive disease [14–16]. In the presence of atherosclerosis, intimal neovascularization arises most frequently from the dense network of vessels in the adventitia, adjacent to a plaque, rather than from the main artery lumen. A strong positive correlation was observed between the degree of adventitial angiogenesis and intimal thickening [17]. During endarterectomy of carotid lesions subintimal hemorrhages and intraplaque hemorrhages are frequently seen in the absence of any visible breach in the intima and the inference is that these are derived from vasa vasorum systems rather than from blood in the vessel lumen.

New blood vessels may have an active role in regulating plaque metabolic activity and actively promoting its growth beyond the critical limits of diffusion from the artery lumen. These new blood vessels, by providing the necessary conduits for the transfer of nutrients, growth factors, and metabolic products, could eventually control the processes that govern plaque progression [12]. These changes in the vasa vasorum are reactive late changes with established plaque. The microvascularity renders the plaque vulnerable to intraplaque hemorrhage and predictable adverse clinical consequence. In other words, the hypothesis is that these changes and events are the end point of an injury and healing cycle and that microintramural hemorrhage from ruptured penetrating branches of the vasa vasorum in high intramural stress points create the nidus that then leads to the whole process of atherosclerosis.

This paper provides an overview of the normal anatomy of vasa vasorum and potential mechanism of plaque formation due to vascular injury (vasa vasorum) and microhemorrhage.

## 2. Vasa Vasorum: Normal Anatomy and Distribution

Accepted mechanism for nourishment of arteries is achieved by diffusion from the lumen of the vessel and from vasa vasorum [18]. In humans, vessels with walls less than 29 smooth muscle cell layers thick normally do not have vasa vasorum [19], and vessels less than 0.5 mm lumen diameter do not have vasa vasorum [20]. Most normal arteries have an extensive network of vasa vasorum in adventitia. In the thoracic aorta, branches of adventitial vasa vasorum penetrate into the media and provide an important source of nourishment. In smaller vessels, such as coronary arteries, vasa vasorum are seen in adventitia but have rarely been imaged in media [21]. However, a study of over one hundred harvested human hearts with silicon infusion of the coronary vessels and video acquisition of flow and distribution showed vasa vasorum feeding penetrating vessels as far as the inner layers of the media [1].

**Figure 1:**
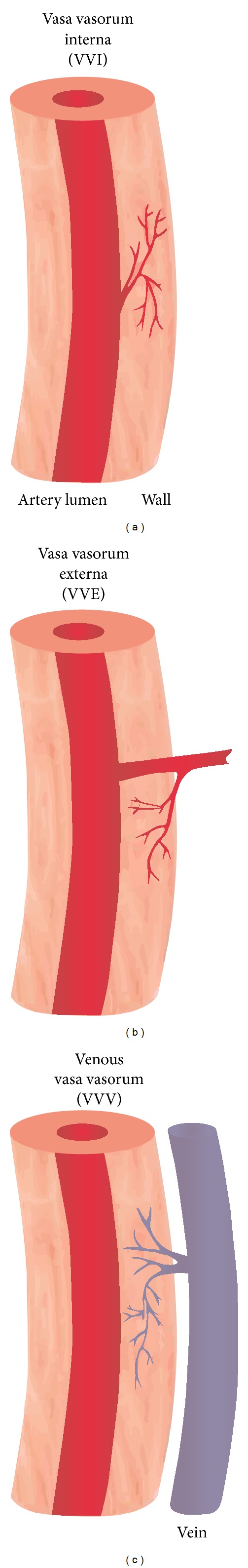
Sketches of the three types of vasa vasorum found in the wall of cow aortae (inspired by Schoenenberger and Mueller [24]). In the Schoenenberger and Mueller study, VVI (a) originated directly from the aorta's main lumen, and VVE (b) originated from intercostal branches deriving from the main lumen and dived back into the aortic wall. VVV (c) developed in the aortic wall and finally drained into branches of concomitant veins (reprint with permission from [25]).

**Figure 2:**
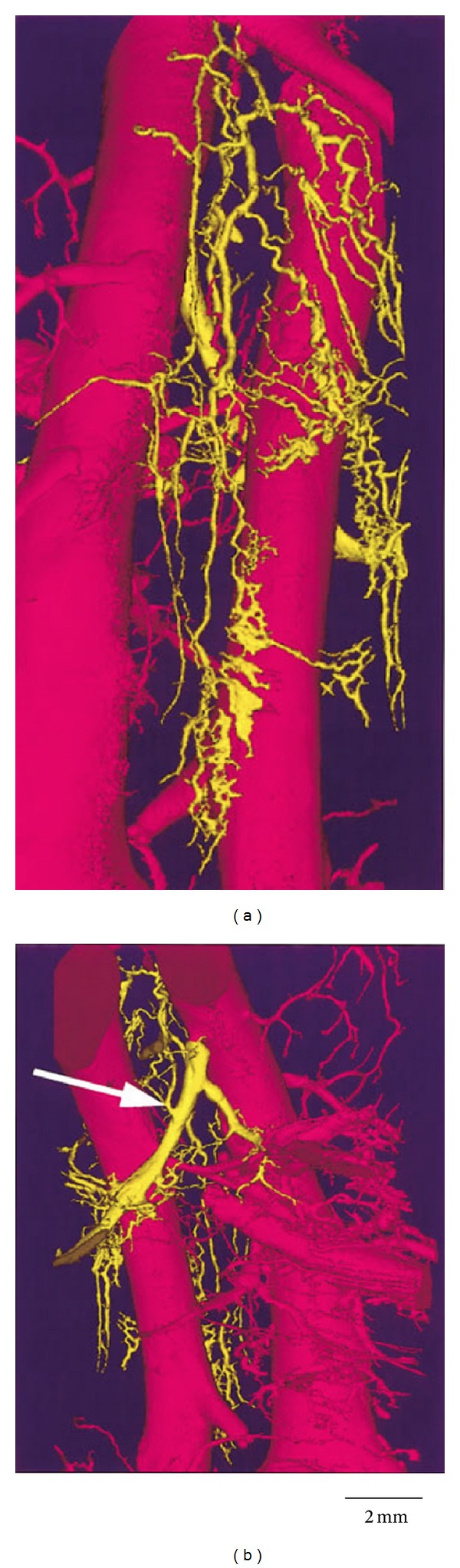
Volume-rendered micro-CT image of a VVV (yellow). (a) In addition to the left anterior descending coronary artery (right, pink) and the concomitant vein (left, pink), the figure shows the complex structure of the VVV, which finally drains into a branch (white arrow) of the concomitant vein ((b), displayed at 180° to (a)). The VVV develops inside the coronary artery wall and forms this complex tree-like structure (reprint with permission from [25]).

Vasa vasorum have tree-like structure, contrary to network in most parts of the body; therefore, vasa vasorum are considered functional end arteries rather than a functional network [22, 23]. Schoenenberger and Mueller described three types of vasa vasorum in the arterial wall based on an animal experiment [24]: the vasa vasorum externa (VVE) and vasa vasorum interna (VVI) supply the arterial wall with oxygenated blood, and the venous vasa vasorum (VVV) drain the arterial wall into concomitant veins (Figure 1). The authors defined the VVE as arising from major branches such as intercostal arteries, and the VVI as originating from the main lumen of the aorta. With the use of micro-CT technology, Gössl et al. for the first time demonstrated both the different types and the fine characteristics of coronary vasa vasorum (Figure 2) [25]. Their results indicated that the branching architecture of the vasa vasorum trees is similar to that of vasculature in general.

From where do the coronary arteries derive their vasa vasorum and blood supply? Barger and Beeuwkes III [1] in their study concluded that the penetrating branches of the vasa vasorum are the pathway of the nourishment of the basement membrane of the intima because no vasa vasorum or vascular channels were demonstrated in this study, emanating from the lumen of the vessel. The coronary vessels are a subset, albeit vitally special, of the arterial tree and approximately the size of medium sized vessels. If the vasa vasorum arise only from the adventitia in the coronary vessels and from the lumen and adventitia in the aorta, this implies a stratification of size of vessel and origin of its blood supply. This knowledge could be decisive in determining which vascular disease results. For example, dissections of the thoracic aorta if the mural blood supply is lumen derived or occlusions of the major and medium sized vessels if vasa vasorum derived. Consider for a moment why iatrogenic breaches in the intima lead to dissections of vessels otherwise prone to atherosclerotic occlusion and rarely if ever spontaneous dissection, whereas the aorta is prone to dissection from intramural exposure to direct luminal pressure by intimal breach and prone to spontaneous dissections—from ruptured VVI.

Galili et al. reported in a pig model a difference in vasa vasorum density throughout different vascular beds in healthy vessels [26]. Using micro-CT, they demonstrated structural heterogeneity of the adventitial vasa vasorum among different vascular beds. Similarly, Hildebrandt et al. demonstrated a significant difference in the vasa vasorum density throughout the arterial system in humans [27]. Their results showed that vasa vasorum density is higher in coronary arteries compared to renal and femoral arteries, supporting the relation between vasa vasorum and the susceptibility to develop atherosclerosis in different vascular beds in humans.

The reason veins and the normal pulmonary artery do not develop atherosclerosis can be explained by the fact that the transmural flux of solute is diminished due to the low venous and pulmonary artery pressures. Furthermore, these vasa vasorum may never get compressed during the entire cardiac cycle due to these lumen pressures being lower than the pressure within the arterial vasa vasorum, thus maintaining adequate flow in the vasa vasorum [28].

## 3. Vasa Vasorum: Association with Plaque Formation and Rupture

The adventitia, media, and the atherosclerotic plaques of the coronary and carotid arteries have their own nutrition supply: the vasa vasorum, a network of small microvessels. When atherosclerosis develops, the vasa vasorum increase including plaque neovascularization, which is thought to play an important role in the progression of atherosclerosis. The extensive vasa vasorum network can function as a conduit for entry of macrophages and inflammatory factors that may promote the progression of angiogenesis and plaque formation. Inhibition of angiogenesis has been shown to reduce macrophages in the plaque and around the vasa vasorum [29].

The vasa vasorum have been the subject of considerable interest for more than a century. The concept that the adventitial vasa vasorum of human coronary arteries play a role in the formation of atherosclerotic plaques dates back to the work of Koester [30] and Winternitz et al. [31] and was revived by Barger et al. [32], who clearly showed in postmortem samples that coronary atherosclerotic segments present a rich vascular network extending from the adventitia to the full thickness of media and intima. It has subsequently been assumed that the density of newly formed vasa vasorum in response to injury is proportional to vessel stenosis [3].

### 3.1. Vasa Vasorum: Association between Vascular Inflammation and Atherosclerosis

Vascular inflammation and atherosclerosis have traditionally been considered an “inside-out” response, which emphasises the central role that inflammatory responses are initiated at the luminal surface [33]. However, there is growing evidence to support a new paradigm of an “outside-in” hypothesis, in which vascular inflammation is initiated in the adventitia and progresses inward toward the media and intima. The functional significance of the adventitia in cardiovascular disease has been explored for many years; however, only in recent years, supporting data are available in the literature to demonstrate inflammation occurring in the adventitia associated with cardiovascular pathogenesis [34–36].

The relationship between the adventitial vasa vasorum and the development of atherosclerosis is further supported by observations in both experimental studies and human subjects. Low incidence of atherosclerosis is associated with a significantly lower vasa vasorum density in animal studies [37, 38]. Intraplaque hemorrhage is common in advanced atherosclerotic lesions, and its occurrence is considered an important event in the manifestation of atherosclerotic disease, causing acute processes such as myocardial infarction as well as cerebrovascular and peripheral acute ischemia [39, 40]. Previous studies have demonstrated the association between plaque neovascularity and quantity of intraplaque hemorrhage [12, 16]. Galili et al. in their study concluded that the microvessels which are smaller in diameter than the first order vasa vasorum are less mature and might be more susceptible to hemorrhage, especially in the presence of hypertension [26]. Gössl et al. in their animal experiments observed significant vasa vasorum neovascularization with higher vascular volume fraction in the proximal left anterior descending in pigs with hypercholesterolemia when compared to the control animals [41]. Furthermore, a link between microvessels and plaque instability was identified in a study consisting of 269 advanced human plaques [42]. Moreno et al. noticed that vessel wall and plaque microvessels are increased in ruptured atherosclerotic plaques, suggesting a contributory role for neovascularization of vasa vasorum in the process of plaque rupture.

### 3.2. Vasa Vasorum: Association between Vasa Vasorum Growth and Plaque Development

Research findings generally support the possibility of correlation between the growth of vasa vasorum, plaque neovascularization, and plaque development [41, 42].* The question remains to be answered, however: what stimulates plaque neovascularization?* Evidence shows that cyclic strain increases tubulogenesis by endothelial cells via various pathways [43, 44]. The cyclic application of strain triggers the endothelial and smooth muscle cells in the arterial wall stimulating neovascularization in the vasa vasorum [45]. Aging of the arteries or stiffening of the arterial wall with increasing age could lead to an increased application of cyclic strain. It is well-known that the compliance of the arteries decreases with increasing age; that is, the arteries get stiffer with age [46]. When the arterial wall stiffens, shear stress on the endothelium by the pulsation might increase displacement of the artery with respect to the surrounding tissue [45]. This results in an increase in shear strain in the adventitial layer that might lead to the vasa vasorum injury and subsequent development of vulnerable plaques.

Many experimental measurements (both* in vivo* and* ex vivo*) have been conducted to determine the parameters of the strain energy correlations, which were found to change significantly through aging, disease, and change in mechanical load [47–50]. Shearing and stress/strain leading to fatigue fractures and intramural hematoma formation from disrupted vasa vasorum are confirmed to be a factor in atherosclerotic development and potentially a significant cause of instability in a plaque leading to an acute ischaemic event. This stress/strain within the arterial wall may represent the most important factor for atherosclerosis with the other well-known risk factors representing accelerators of atherosclerosis [51].

## 4. Summary and Conclusion

In summary, there is increasing evidence showing the association between the distribution and density of vasa vasorum in the arterial wall and severity of plaque formation, as well as the development of atherosclerosis. However, further studies are needed to clarify whether the vasa vasorum play a causative or only reactive role in the atherogenesis. Various imaging modalities are used not only to demonstrate normal anatomy of vasa vasorum, but also characterize the composition, burden and neovascularization of atherosclerotic plaque associated with vasa vasorum injury. This will be discussed in the paper of imaging modalities in the visualization of vasa vasorum and atherosclerotic plaque.
